# Structure of *Trypanosoma brucei* glutathione synthetase: Domain and loop alterations in the catalytic cycle of a highly conserved enzyme

**DOI:** 10.1016/j.molbiopara.2009.12.011

**Published:** 2010-04

**Authors:** Paul K. Fyfe, Magnus S. Alphey, William N. Hunter

**Affiliations:** Division of Biological Chemistry and Drug Discovery, College of Life Sciences, University of Dundee, Dow Street, Dundee, DD1 5EH, United Kingdom

**Keywords:** AMP-PNP, adenylyl imidodiphosphate, GS, glutathione synthetase, GSH, glutathione, HEPES, 4-(2-hydroxyethyl)piperazine-1-ethanesulfonic acid, N-(2-hydroxyethyl)piperazine-N-(2-ethanesulfonic acid), MOPS, 3-(N-morpholino)-propanesulfonic acid, NCS, non-crystallographic symmetry, *Tb*, *Trypanosoma brucei*, TEV, tobacco etch virus, TLS, translation/libration/screw, TSA, trypanothione synthetase, T[SH]_2_, trypanothione, ATP-grasp, Glutathione, Glutathione synthetase, *Trypanosoma brucei*, Trypanothione, X-ray structure

## Abstract

Glutathione synthetase catalyses the synthesis of the low molecular mass thiol glutathione from l-γ-glutamyl-l-cysteine and glycine. We report the crystal structure of the dimeric enzyme from *Trypanosoma brucei* in complex with the product glutathione. The enzyme belongs to the ATP-grasp family, a group of enzymes known to undergo conformational changes upon ligand binding. The *T. brucei* enzyme crystal structure presents two dimers in the asymmetric unit. The structure reveals variability in the order and position of a small domain, which forms a lid for the active site and serves to capture conformations likely to exist during the catalytic cycle. Comparisons with orthologous enzymes, in particular from *Homo sapiens* and *Saccharomyces cerevisae*, indicate a high degree of sequence and structure conservation in part of the active site. Structural differences that are observed between the orthologous enzymes are assigned to different ligand binding states since key residues are conserved. This suggests that the molecular determinants of ligand recognition and reactivity are highly conserved across species. We conclude that it would be difficult to target the parasite enzyme in preference to the host enzyme and therefore glutathione synthetase may not be a suitable target for antiparasitic drug discovery.

## Graphical abstract

The close similarity of *Trypanosoma brucei* glutathione synthetase to the human orthologue indicates that the enzyme would be a difficult target for drug discovery.

## 1 Introduction

The tripeptide glutathione (l-γ-glutamyl-l-cysteinyllycine, GSH) is an abundant non-protein thiol implicated in a variety of cellular functions. The majority of organisms use GSH, in conjunction with glutathione reductase and a glutathione peroxidase, to regulate intracellular thiol levels, to control redox metabolism and therefore protect against reactive oxygen species. GSH also contributes to the metabolism of drugs, carcinogens, and in maintaining cysteine residues in their reduced form [1,2].

Trypanosomatids are unusual in that they derive protection against oxidative damage and maintain intracellular thiol redox balance through the utilization of trypanothione, a peptide–polyamine conjugate (*N*^1^,*N*^8^-bis(glutathionyl)spermidine, T[SH]_2_) [3–5]. This protective mechanism requires the presence of a unique peroxidase pathway comprising trypanothione reductase, tryparedoxin and tryparedoxin peroxidase. The divergence from the human host in this aspect of biology is of basic interest and we have determined the structure–function relationships for the tryparedoxin peroxidase pathway components [6–10]. The protective peroxidase pathway is recognized as a potential route for the development of urgently sought antiparasitic drugs [4,5]. It is also recognized that disruption of T[SH]_2_ biosynthesis might represent a useful strategy in this respect [11] and characterization of the component enzymes is an important first step in assessing the potential of such targets [12].

**Fig. 1:**
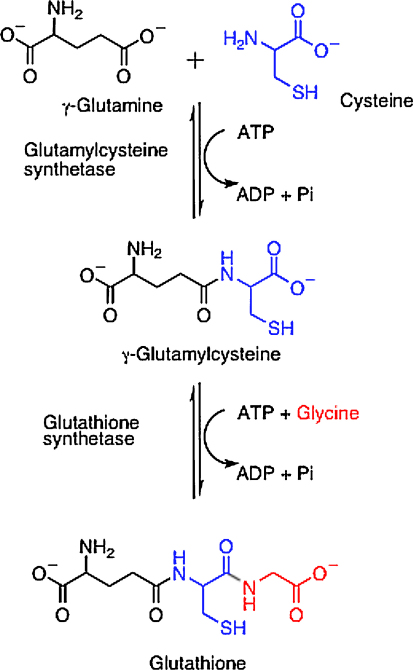
Biosynthesis of glutathione.

Biosynthesis of the peptide component of T[SH]_2_ involves two enzymes. First glutamylcysteine synthetase is responsible for the ligation of cysteine to glutamate and then glutathione synthetase (GS) converts the dipeptide to GSH by addition of glycine (Fig. 1). The bifunctional enzyme trypanothione synthetase-amidase catalyses the stepwise addition of two molecules of glutathione (GSH) onto spermidine and is also capable of hydrolyzing T[SH]_2_ and/or glutathionylspermidine back to spermidine and glutathione [13,14].

We set out to characterize the structure of *Trypanosoma brucei* glutathione synthetase (*Tb*GS) and to assess its potential as a target for design, or discovery, of a novel therapeutic agent for treatment of African trypanosomiasis. We now report the preparation of an efficient bacterial protein production system, a purification protocol, and a low resolution (3.15 Å) crystal structure. Comparisons with previously determined structures of GS from *Homo sapiens* (*Hs*GS) and *Saccharomyces cerevisae* (*Sc*GS) [15,16] have been carried out.

## 2 Materials and methods

### 2.1 Protein preparation and crystallization

The gene encoding *Tb*GS was amplified from genomic DNA using the primers 5′-catatgGTGTTAAAATTGTTGCTG and 5′-ctcgagTTACGCTACAACCGCTAA (lower case sequences correspond to the restriction sites used for cloning—Nde1/Xho1). Following TOPO cloning (Invitrogen) of the polymerase chain reaction product, the gene was ligated into a pET15b (Novagen) expression vector modified to encode a Tobacco Etch Virus (TEV) protease cleavage site. The resulting plasmid was transformed into *Escherichia coli* Rosetta (DE3) pLysS. Cultures were grown at room temperature in autoinduction medium [17] containing 50 μg/ml ampicillin and 12 μg/ml chloramphenicol for two days, and cells harvested by centrifugation. The pellet was resuspended in 25 mM HEPES pH 7.5, 500 mM NaBr, 2 mM β-mercaptoethanol, 1 mM MgCl_2_, and 5 mM imidazole, and cells lysed using a French Press. Cell debris was removed by centrifugation at 40,000 × *g*.

*Tb*GS was purified by nickel affinity chromatography on a 5 mL HisTrap column (GE Healthcare) using an imidazole gradient. Following overnight dialysis at 4 °C into 25 mM HEPES pH 7.5 and 250 mM NaBr, gel filtration chromatography using a Superdex200 26/60 column (GE Healthcare) completed the purification. Gel filtration indicated that in solution *Tb*GS exists as a dimer of approximate mass 120 kDa. The high level of purity (>95%) was confirmed by sodium dodecyl sulfate–polyacrylamide gel electrophoresis and matrix-assisted laser desorption/ionization-time of flight mass spectrometry. The sample was dialysed into 50 mM MOPS pH 7.0 and 100 mM NaBr then concentrated to 6 mg mL^−1^ using a Vivaspin 20 (Sartorius) to provide a stock solution for crystallization. The yield was 8 mg of purified *Tb*GS per litre of bacterial culture.

*Tb*GS was crystallized by the hanging drop vapour diffusion method. Protein was prepared to a final concentration of 6 mg mL^−1^ with 4 mM adenylyl imidodiphosphate (AMP-PNP), 4 mM GSH, 2 mM dithiothreitol and 2 mM MgCl_2_. A drop containing 1 μL of this protein mixture was mixed with 1 μL of reservoir containing 14% polyethylene glycol 4000, 100 mM citrate buffer pH 5.4, and 200 mM (NH_4_)_2_SO_4_. Crystals, orthorhombic blocks, grew within a few days at 20 °C. They were first characterized in-house using a Rigaku 007 rotating anode X-ray generator coupled to a RAXIS IV^++^ image plate detector, cryoprotected by soaking for 5 s with mother liquor substituted with 15% ethylene glycol and diffracted to 3.5 Å. The crystals were stored in liquid N_2_ for use at the European Synchrotron Radiation Facility (ESRF, Grenoble, France) for data collection.

### 2.2 X-ray data collection, processing, structure solution and refinement

Diffraction data were measured on beamline ID14-4 of the ESRF using an ADSC Quantum-4 charge coupled device detector. Data were processed using XDS and scaled using XSCALE [18]. Data statistics are summarised in Table 1. The structure was solved by molecular replacement using the program PHASER [19] with the structure of *Sc*GS [1M0W; 16] as the search model. Four molecules (two dimers) were located per asymmetric unit, giving a *Z*-score of 54.2. Refinement was performed using REFMAC5 [20] utilizing both non-crystallographic symmetry (NCS) and Translation/Libration/Screw (TLS) refinement, alternating with rounds of manual inspection and re-building in COOT [21] with the inclusion of a few water molecules, several well-defined sulfates and, in each active site a molecule of GSH. MOLPROBITY [22] was used to investigate the model geometry along with the validation tools within COOT. Figures were prepared using PyMOL [23]. Interface region calculations were performed using the PISA server [24], and sequence alignments calculated using MUSCLE [25]. The coordinates and structure factor data have been deposited with the Protein Data Bank (PDB) under the accession code 2wy0. Unless stated otherwise, discussions relating to the GS structure from *S. cerevisae* are based on PDB entry 1M0W, a model determined to a resolution of 1.8 Å. The *Hs*GS structure discussed is from PDB entry 2HGS; a structure reported at 2.1 Å resolution and the *E. coli* GS structure is from PDB entry 1GSA; reported at 2.0 Å resolution.

## 3 Results and discussion

### 3.1 General comments

The structure of *Tb*GS was solved by molecular replacement using the structure of the yeast enzyme, *Sc*GS [16] as the search model. The refinement statistics and model geometry (Table 1) indicate that the refinement has produced a low-resolution model of acceptable quality. Four molecules are present in the asymmetric unit, forming two dimers consisting of subunits A with B, and C with D. The subunits are very similar in structure with r.m.s.d. values of 0.9 Å (A–B), 0.9 Å (A–C) and 0.8 Å (A–D) when they are superimposed with SSM [26]. This is in part a consequence of the application of NCS during the refinement. Subunit A has five disordered sections (residues 131–132, 178–181, 234–248, 444–455, and 537–544), subunit B has four (residues 128–137, 234–246, 401–406, and 426–464), subunit C has seven (residues 127–134, 181–184, 238–246, 414–416, 423–430, 442–464, and 536–543) and subunit D has three (residues 128–132, 235–245, and 425–464). In general the following discussions will deal with molecule A, it being the most complete of the four subunits in the asymmetric unit. There are several regions in the other subunits, which were better defined than in molecule A and these will be discussed as appropriate.

Superimposition of the *Tb*GS structure on the other available GS enzyme structures revealed strong structural conservation with the *S. cerevisae* (31% sequence identity) and human enzymes (31% sequence identity). These gave r.m.s.d. values of 1.7 Å and 1.6 Å respectively. The *E. coli* enzyme, which has only 17% sequence identity, shows significant differences to these with an r.m.s.d. value of 3.2 Å when superimposed on the *Tb*GS structure. Description of the overall structure of *Tb*GS will, therefore, follow closely previous descriptions of GS and we maintain a nomenclature already defined for these structures [15,16,27–29].

The modest resolution to which diffraction data have been acquired necessarily limits the accuracy of the model and a conservative approach has been adopted in reporting structural details. We have assigned hydrogen bonding and salt bridge interactions in general if the distance between relevant functional groups falls in the range 2.5–3.5 Å.

### 3.2 Overall structure

**Fig. 2:**
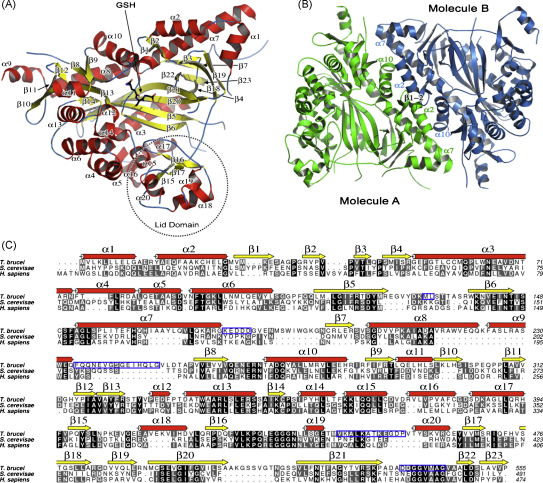
Structure of *Tb*GS. (A) Overall structure of a subunit and position of GSH. (B) Structure of the *Tb*GS dimer, with those secondary structure elements identified to be involved in the interface marked. (C) Sequence alignment of *Tb*GS with the sequences for *Sc*GS and *Hs*GS. The assigned secondary structure of *Tb*GS is indicated, while those regions not resolved by well-ordered electron density (for subunit A) are enclosed in blue boxes. Strictly conserved residues are enclosed in black. Residues conserved in two out of three sequences are enclosed in grey. (For interpretation of the references to color in this figure legend, the reader is referred to the web version of the article.)

The structure of subunit A is presented in Fig. 2A, colored according to secondary structure. The *Tb*GS subunit is composed of two domains, a large “core” domain and a smaller “lid” domain (Fig. 2A) that together form an ATP-grasp fold [30,31]. The core domain is itself constructed from two sub-domains placed on either side of a four-stranded anti-parallel β-sheet formed by β5, β6, β20 and β21. One of the sub-domains comprises four parallel and two anti-parallel β-strands enclosed by short α-helices, for example helices 9, 10, 11, 12, 13, 14, and 15 (Fig. 2A). The second sub-domain consists of three helices (α1, α2, and α7) positioned beside a four-stranded anti-parallel β-sheet (β3, β7, β18 and β19). The lid domain is poorly resolved in the electron density map, with only a short section of anti-parallel β-sheet observed in subunits B, C and D. Subunit A displayed greater order in this region, allowing for model building of all but a short sequence between residues 443 and 446. Secondary structure within the lid domain of subunit A can be assigned with confidence, however the positioning of some side chains was less clear. The fold of this small domain is similar to that observed in other GS structures, with a core of three β-strands forming one wall and lid of the active site with a further three α-helical sections exposed on the protein surface (Fig. 2A).

**Fig. 3:**
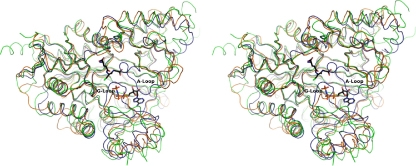
Stereoview showing the superimposed main chain trace of *Tb*GS, *Sc*GS and *Hs*GS. *Tb*GS is in green, *Hs*GS (Protein Databank code 2HGS) in blue and the *Sc*GS structure (Protein Databank code 1M0W) in orange. The approximate positions of the A- and G-loops as positioned in the closed *Hs*GS structure are labeled. The orange *Sc*GS structure appears to represent an intermediate state with the *Tb*GS giving the most open structure, *Hs*GS the closed structure. The A-loop is not resolved in *Tb*GS. (For interpretation of the references to color in this figure legend, the reader is referred to the web version of the article.)

The lid domain appears to be inherently more mobile than the remainder of the enzyme as indicated by the less ordered electron density and elevated thermal parameters in the model. The function of this lid is to fold down on top of, or grasp, the bound ATP. Disorder of the lid domain has been observed previously in GS [15,16,29] and in related ATP-grasp fold enzymes such as biotin carboxylase [32]. Despite attempts to co-crystallize *Tb*GS with ATP or the non-hydrolysable AMP-PNP, these ligands were not observed. The absence of a ligand in the ATP-binding site may, in part, explain the problems encountered when attempting to interpret diffuse electron density for this region of the molecule. It should be noted, however, that *Tb*GS contains two insertions within this region of four and six residues (Fig. 2C), a point discussed later. The importance of the lid domain in ATP-grasp enzymes is not simply to restrict the movement of ligand bound prior to catalysis, or aid in the orientation of the ligands, but also to prevent the intrusion of solvent into the active site, since the phosphate intermediates formed as part of the reaction mechanism are vulnerable to hydrolysis [15]. In *Tb*GS, the lid domain in subunit A is considered to be in an open position (Fig. 3). Comparison of the positioning of the lid domain in both *Sc*GS and *Hs*GS when nucleotide is bound reveals much greater coverage of the top of the active site than observed in *Tb*GS (Fig. 3).

Size exclusion chromatography indicated that *Tb*GS is a dimer in solution, and the asymmetric unit was found to comprise two such dimers, subunits A–B and C–D. The *Tb*GS dimer is formed in a similar manner to that observed for the *Sc*GS and *Hs*GS with a non-crystallographic twofold rotation axis relating the subunits to each other (Fig. 2B). The subunit–subunit interface was calculated to cover ∼1820 Å^2^ or 7.5% of the total surface area of a subunit, and is formed through contacts from a short span of anti-parallel β-sheet (β1–2) and contacts from α2 and a short section of α7 along with α10. Salt bridge interactions are formed between Glu12 and Arg288, Arg13 and Glu292, Arg176 and Asp251 plus both Lys21 and His164 are in a position to interact with Glu279 (data not shown). A short stretch of anti-parallel β-sheet between adjacent β19 (residues 486–490), along with salt bridge interactions between Arg491 and Glu16 form the crystal contacts between dimers within the asymmetric unit (data not shown).

The sequence alignment of *Tb*GS with *Sc*GS and *Hs*GS reveals six insertions of greater than four residues in the trypanosomatid enzyme (Fig. 2C). The first two of these, a six-residue insertion within α7 and eight residues inserted before β7, combine to form an elongated loop compared to the yeast and human enzymes. The third and fourth insertions occur on one side of the *Tb*GS core domain formed by helices α8 and α9. They produce an extension to α8 compared to the *Sc*GS and *Hs*GS structures in addition to a helix (α9) unique to *Tb*GS. Residues 238–245 have not been resolved in the *Tb*GS electron density maps, but the structure becomes ordered at the structurally conserved β8. The fifth insertion, just prior to α20, is part of the lid domain and is largely disordered, but again it would appear to form an extended loop. Finally, a nine-residue insertion forms an elongated twisted β-turn between β20 and β21. All of these insertions occur on the surface of GS distant from the active site, substrate/cofactor binding sites and the dimerization interface. An alignment of trypanosomatid GS sequences with those from other eukaryotic species (data not shown) reveals these insertions are mainly restricted to the trypanosomatid enzymes. An exception is found in GS from the mosquito *Anopholes gambiae* where it is noted that insertions 1 and 2 are present although there is no appreciable sequence homology in these segments of the proteins.

### 3.3 Active site structure and ligand binding

**Fig. 4:**
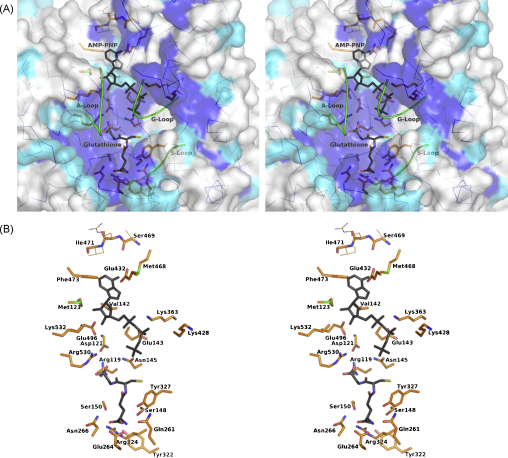
Stereoviews of the active site. A molecule of GSH is bound in the active sites of all four subunits in the asymmetric unit of *Tb*GS. GSH is depicted as a stick model colored by atom type (C: black, O: red, N: blue, and S: yellow). A molecule of ATP is shown in a similar fashion (all black). The position of ATP was derived from the structure of the *Sc*GS:AMP–PNP complex (Protein Databank code 1M0W) overlayed on *Tb*GS. (A) *Tb*GS is shown as van der Waals surface colored according to the conservation of the residues in and around the active site (as derived from the alignment in Fig. 2C), with blue showing 100% identity and cyan where an identical residue is found in two out of the three sequences. Two green loops mark the anticipated position of part of the A-loop, missing in the *Tb*GS structure, and the position of the S-loop. A third green loop shows the position of the G-loop in the lid domain as observed in *Hs*GS (Protein Databank code 2HGS). (B) The positions of key residues discussed in the text contributing to binding of GSH and ATP. (For interpretation of the references to color in this figure legend, the reader is referred to the web version of the article.)

**Fig. 5:**
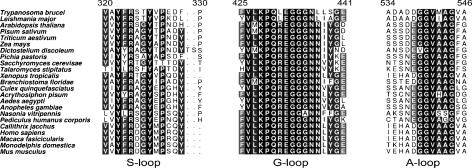
The alignment of the S-, G- and A-loops from 23 eukaryotic GS sequences. While the G-loop shows identity approaching 100% the other two loops show lower, yet still significant levels of conservation. The numbering relates to the sequence of *Tb*GS.

*Tb*GS has an ATP-grasp fold that carries a well-defined nucleotide-binding site. The floor of the nucleotide-binding pocket is formed by the anti-parallel strands β5 and β6 with walls formed on one side by strands β20 and β21 and on the other by the lid domain. The GSH binding site is positioned over the top of a loop linking β6 to α7, with further interactions to residues from the loops that link β8 to α10 and β13 to α12 (Fig. 2A). Sequence alignments of the ATP-grasp family in conjunction with available structural data have highlighted three flexible loops regions as being important for the activity of these enzymes [31]. These flexible segments are known as the substrate binding loop (S-loop), the glycine rich loop (G-loop) and the alanine rich loop (A-loop) [15,28]. The positions of these loops, together with most of the residues that interact with GSH or are predicted to bind ATP are shown in Figs. 3 and 4, with an alignment of these regions in 23 eukaryotic GS enzymes presented in Fig. 5.

The S-loop extends from β13 and lies across the top of the bound GSH. The conservation is lower in the S-loop when compared to the G- and A-loops but three residues important for interaction with ligands are conserved. Tyr322 aids the orientation of Arg324 that interacts with the glutamyl carboxylate of GSH. Tyr327, held in position by a hydrogen bond donated from Gln261 (conserved as glutamine or glutamate in other GS sequences) lies across the face of the cysteinyl moiety of GSH. The presence of the aromatic side chain may afford some protection against side reactions occurring with the reactive thiol group.

The A-loop is in close proximity to the glycyl end of GSH and interacts using main chain functional groups. The amides of Val541 and Met542 donate hydrogen bonds to the glycyl carboxylate group (data not shown). This carboxylate group also accepts a hydrogen bond donated from the side chain of the Arg530 (conserved as Arg450, Arg467 in *Hs*GS and *Sc*GS respectively). In *Hs*GS, GSH displays a similar interaction pattern with the A-loop, although the residues concerned are Val461 and Ala462 [15]. The amino acid type at the latter position varies in the sequences from the most common alanine to low occurrences of serine, isoleucine, histidine and methionine. The presence of two glycine residues preceding this section is almost completely conserved in eukaryotic GS sequences (valine in a single sequence, *Dictyostelium discoideum*, see Fig. 5), as are the two residues that follow; usually alanine, otherwise serine or glycine, and a completely conserved glycine. The sequence alignments alone suggest that *Sc*GS would show a high level of structural similarity with *Hs*GS and *Tb*GS in this region. However, this is not the case. In *Sc*GS the loop in this region extends away from the GSH binding site or is disordered. Neither of the reported *Sc*GS structures possesses a complete molecule of GSH in the active site with only γ-glutamylcysteine being observed. The lack of the glycyl moiety might preclude conformational changes to lock down and order residues in this part of the active site as observed for *Hs*GS and *Tb*GS.

The G-loop, between β16 and α19, is a highly conserved region with eight residues out of twelve found to be identical in the sequences examined. The remaining four residues involve conservative changes (Fig. 5). This loop was most problematic to model and analyze, lying as it does within the flexible lid domain. In *Tb*GS the loop has been successfully modeled in subunit A with a partial model in subunit C. The loop contains three conserved glycines, one of which has previously been assigned as having a role in stabilizing the pentavalent phosphate intermediate during the phosphorylation step of the catalytic cycle [15,16]. While this section is modeled in *Tb*GS, the placement of the glycines is distant from the expected position over the active site. Previous structural studies of GS have required AMP-PMP or ADP to be bound within the nucleotide-binding site before the G-loop can be resolved, and this binding appears to cause significant reorganization of the lid domain. The structures of *Sc*GS revealed that binding of AMP-PNP caused a 64° rotation [16,33] and maximal shift of some 24 Å of this section of the molecule relative to the core domain.

Away from the A- and G-loop regions there are several other interactions observed between GSH and *Tb*GS (Fig. 4B). The γ-glutamyl moiety forms salt bridge interactions with Arg324 and hydrogen bonds to Ser150, Glu264 and Asn266. These residues are strictly conserved in *Hs*GS as Arg267, Ser151, Glu214 and Asn216; and in *Sc*GS as Arg285, Ser153, Glu228 and Asn230. The cysteine carbonyl and amide groups potentially form hydrogen bonds to Arg119 and Ser148 respectively. This pair of residues is strictly conserved as Arg125, Ser149 in *Hs*GS and Arg128, Ser151 in *Sc*GS. The glycinyl carboxylate is placed to form salt bridge interactions with *Tb*GS Arg530 and again the interaction appears to be strictly conserved in *Hs*GS with Arg450 and in *Sc*GS with Arg467.

In general, the part of the ATP-grasp enzyme family active site that binds the nucleotide cofactor is well-ordered and does not change conformation upon ligand binding [14]. Therefore, on the basis of sequence and structural homology we expect ATP to bind *Tb*GS in a similar manner to that discussed for other ATP-grasp proteins and a model of nucleotide binding, primarily based on the *Sc*GS:AMP–PNP complex structure [16] is presented in Fig. 4B. The adenine would make a large number of hydrophobic interactions with *Tb*GS Met123, Val142, Met468, Ile471 and Phe473. These five residues are highly conserved in *Hs*GS (Met129, Ile143, Met398, and Ile401) and *Sc*GS (Leu132, Val145, Met415, and Ile418). A potential hydrogen bond network can also be identified with adenine N6 donating to the carbonyl of Ser469 and adenine N1 accepting from the amide of Ile471. Lys532, Glu496 and Glu432 are in a position such that they would be capable of forming hydrogen bonds to the ribose group of ATP. The binding and orientation of the cofactor phosphates would be expected to involve Lys363 and Lys428, matching to Lys305 and Lys364 of *Hs*GS, and also the Lys324, Lys382 pair in *Sc*GS. However, in the *Tb*GS:ATP model only the first lysine residue matches the position found in the other species. The latter, Lys428, is pointing out of the active site due to the orientation of the G-loop in the *Tb*GS structure. We predict a significant re-organization of the G-loop would accompany nucleotide binding and therefore an interaction with Lys428 may well be conserved. The phosphate tail of ATP would be expected to bind near, interacting with residues on the highly conserved G-loop. These residues are not shown in Fig. 4B since in the *Tb*GS structure the G-loop, which is part of the lid domain, is in an open conformation.

Two magnesium ions stabilize the position of the phosphate tail of the cofactor in the active site of ATP-grasp enzymes and are thought to influence the stability of the reaction intermediates during catalysis. The positions of such cations are clearly observed in the *Sc*GS:AMP–PNP complex structure where four acidic residues either coordinate the Mg^2+^ ions directly or bind coordinating water molecules in conjunction with direct phosphate-cation coordination [16]. In *Sc*GS these residues are Asp130, Glu146, Glu386 and Glu442. All four residues are strictly conserved in *Tb*GS and *Hs*GS (Fig. 2C). In *Tb*GS, three of these residues, Asp121, Glu146 and Glu496 are placed to fulfill the same role. The fourth residue, Glu442 is observed to be distant from the cofactor-binding site since the mobile lid domain is in an open conformation. Our model indicates also that Arg119 and Arg530 of *Tb*GS, residues important for substrate recognition by interaction with glycine and cysteine moieties respectively, and catalysis as discussed previously, are structurally conserved.

### 3.4 Concluding remarks

We have determined a low-resolution crystal structure of the parasite enzyme *Tb*GS in complex with the product GSH. The structure indicates different conformations of the lid domain with respect to the core domain of this enzyme. Our analysis of the structure, sequence and structure comparisons with the enzymes from the host, *Hs*GS and *Sc*GS are reported. Part of our motivation to characterize *Tb*GS was to investigate the potential of this enzyme as a target for therapeutic intervention against African trypanosomiasis. We note a high degree of structural similarity between *Tb*GS and orthologues with conservation of the amino acids that create the enzyme fold, form the active site, bind to the cofactor ATP, the substrate and that participate in catalysis. Structural differences that are observed are ascribed to the different conformations of the enzyme depending on the presence or absence of ligands in the active sites. The high degree of conservation in the active sites would render it difficult to identify a potent species-specific inhibitor of value in the development of new therapeutic agents.

## Figures and Tables

**Table 1:** Crystallographic statistics.

Space group	*P*2_1_2_1_2_1_
Unit cell lengths (Å)	92.5 125.0 242.9
Maximum resolution (Å)	3.15
Unique reflections	49689
Completeness (%)	99.9 (100)^a^
$\langle I/\sigma (I)\rangle$	9.0 (2.7)
Mosaicity (°)	0.4
Redundancy	4.0 (4.1)
*R*_merge_^b^	11.6 (50.6)
Refinement statistics	
Resolution range (Å)	39.5–3.15
No. of reflections	47141
*R*_work_^c^	21.5
*R*_free_^d^	28.8
Protein residues	1996
Ligands	4 GSH, 7 sulfates, 22 waters

r.m.s. deviations from ideal geometry	
Bond lengths	0.012
Bond angles	1.5

*B* values (Å^2^)	
From Wilson plot	77.9
Mean B over all atoms	68.4
Ramachandran favoured/allowed/outliers^e^ (%)	90.4/8.2/1.4

a Values in parentheses refer to the highest resolution bin (3.32–3.15 Å).

b aa $R_{\text{merge}}=\sum h\sum i||(h,i)-\langle I(h)\rangle \sum h\sum iI(h,i)$.

c *R*_work_ = *∑h* *k* *l*||*F*_o_| − |*F*_c_||/*∑|F*_o_*|,* where *F*_o_ is the observed structure factor amplitude and the *F*_c_ is the structure-factor amplitude calculated from the model.

d *R*_free_ is the same as *R*_work_ except determined using 5%, of the data that are not included in any refinement calculations.

e Ramachandran analysis performed using MolProbity [22].
